# Comparative Tear Fluid Proteomics to Explore Treatment Responsiveness in Diabetic Macular Edema: A Pilot Study

**DOI:** 10.1021/acs.jproteome.5c00181

**Published:** 2025-08-29

**Authors:** I-Hsin Ma, Yuan-Ling Hsu, Hsin-Yi Wu, Yi-Ting Hsieh, Szu-Hua Pan

**Affiliations:** † Department of Ophthalmology, 63423 National Taiwan University Hospital, Hsinchu Branch, Hsinchu 302, Taiwan; ‡ Graduate institute of medical genomics and proteomics, 38005 National Taiwan University College of Medicine, Taipei 100, Taiwan; § Instrumentation Center, 33561 National Taiwan University, Taipei 106, Taiwan; ∥ Department of Ophthalmology, National Taiwan University Hospital, Taipei 100, Taiwan; ⊥ Ph.D Program in Translational Medicine, National Taiwan University and Academia Sinica, Taipei 115, Taiwan; # Genome and System Biology Degree Program, College of Life Science, National Taiwan University, Taipei 106, Taiwan

**Keywords:** diabetic retinopathy, diabetic macular edema, antivascular endothelial growth factor, tear fluids, proteome, differentially expressed proteins

## Abstract

Diabetic macular edema (DME) is a leading cause of vision loss in patients with diabetes, with variable responses to intravitreal antivascular endothelial growth factor (VEGF) therapy. This study aimed to identify tear fluid proteins linked to treatment responsiveness. Tear fluid samples were collected from 13 patients with diabetes (21 eyes with DME), among which 9 samples were analyzed using tandem mass tag-labeled liquid chromatography–mass spectrometry, and all 21 samples were validated with ELISA. Protein identification and quantification were performed using MaxQuant. Gene set enrichment analysis identified differentially expressed proteins, and an enzyme-linked immunosorbent assay was used to validate specific protein targets. Among the 3121 quantified proteins, DME-related proteins were enriched in cellular localization, oxidative stress response, and VEGFA-VEGFR2 signaling. Responders’ tear fluid proteomes were linked to modulation of the extracellular matrix and inflammation, whereas nonresponders showed enrichment in inflammation and NOTCH/WNT pathways. Targeted protein analysis demonstrated that the interplay between TIMP1, MMP9, and insulin growth factor binding protein 3 (IGFBP3) correlated with treatment response. These results suggest that tear fluid proteomic analysis is a promising alternative to intraocular sampling for evaluating DME responsiveness, offering insights consistent with aqueous and vitreous proteomes while aligning with clinical prognosis and disease pathophysiology.

## Graphical abstract

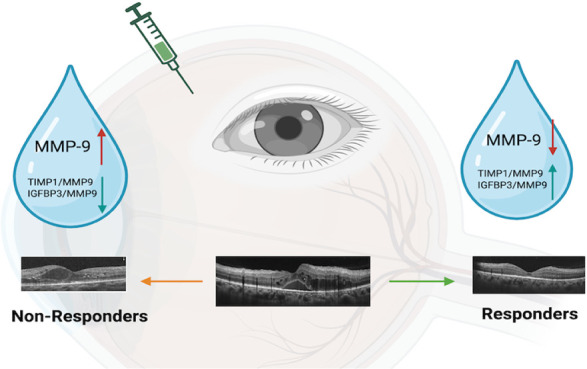

## Introduction

The treatment of diabetic macular edema (DME) has considerably evolved over the years, shifting from laser therapy to intravitreal injection-based therapy using antivascular endothelial growth factor (VEGF) agents and steroids.^1–4^ The use of anti-VEGF therapy as the first-line treatment for center-involved diabetic macular edema (DME) is globally recognized and endorsed. This consensus is also reflected in Taiwan. In circumstances where three to five injections fail to produce an adequate therapeutic response, it is advisible to explore second-line interventions. Such interventions may comprise intravitreal steroid implants, macular grid laser photocoagulation, or surgical vitrectomy, as appropriate to the clinical context.^5^ However, DME resolution from anti-VEGF ranges from 30%–70%, retaining some 40%–50% chronic persistent edema.^6–8^ Identifying patients who do not respond well to anti-VEGF therapy can result in earlier initiation of adjunctive steroid or macular grid treatment, ultimately helping maintain improved vision.

Several biomarkers have been investigated in relation to diabetic retinopathy severity, primarily in the aqueous or vitreous humor, owing to their intraocular nature. Recently, proteomic data on DME have been released, providing further insights into potential DME biomarkers.^9,10^ Among these studies, aqueous proteomics has demonstrated that proteins associated with transportation, coagulation, and inflammatory responses are upregulated with the increased severity of diabetic retinopathy (DR).^11^ Some of the specific proteins found to be substantially altered in DME include phosphatidylethanolamine-binding protein 1, parkinsonism-associated delicate, crystallin gamma C/S, and crystallin beta B2.^9^ Furthermore, targeted cytokine analysis in an aqueous medium has revealed increased levels of interleukin (IL)-2, IL-8, platelet growth factor, and VEGF in DME, with IL-8 being the only cytokine associated with the responsiveness to anti-VEGF therapy.^12,13^

In studies on proteins found in the vitreous humor, the expression of Cathepsin B, D, and L was found to be downregulated in proliferative diabetic retinopathy (PDR). This result was further confirmed by *in vitro* studies on human retinal endothelial cells (hREC).^14^ In DME, the most abundant proteins are hemopexin, beta-crystallin S, clusterin, and transthyretin.^15^ Another study focusing on DME in the vitreous revealed an increase in protein pigment epithelium-derived factor, apolipoprotein (Apo)­A-4, ApoA-1, thyroid receptor-interacting protein 11, and the putative regulatory delta subunit of the cyclic nucleotide phosphodiesterase delta from mammalian photoreceptors.^16^

In addition to detecting alterations in intraocular fluid, tear fluid has been identified as a potential target for analysis. As the most readily accessible and minimally invasive ocular fluid, tear fluid play an important role in different eye diseases.^17^ A study of tear fluid proteomics for DR showed proteins such as lipocalin 1, lactotransferrin, lacritin, lysozyme C, lipophilin A, and immunoglobulin lambda chain, which are linked to disease severity.^18^ However, a tear fluid proteomics study on the responsiveness of DME to anti-VEGF treatment has not yet been conducted. Identifying the molecular differences in different disease statuses could improve clinical evaluations and guide the development of more suitable treatments, ultimately maximizing visual gain and maintaining functional vision in patients with DME. Previously, we have set up our tear fluid proteome pipeline and confirmed proteomic differences between DME and nondiabetic controls.^19^ Thus, our study aimed to compare the tear fluid proteomics between responders and nonresponders to anti-VEGF treatment in a population with DME.

## Experimental Section

### Subjects’ Enrollment

Participants were recruited based on their diabetes status and the presence of eye involvement. Our focus was on individuals with diabetic macular edema (DME), irrespective of the stage of diabetic retinopathy (DR). The inclusion criteria were as follows: 1) central retinal thickness >300 μm, 2) visual acuity ranging from Snellen 0.05 to 0.8, 3) a willingness to undergo anti-VEGF treatment as the initial therapy. Furthermore, all participants had to be treatment naïve, meaning they had not previously received any forms of DME treatment, namely, including macular grid laser therapy, anti-VEGF injections, or intravitreal steroid injections. All enrolled patients were >20 years old and provided signed informed consent.

Besides exclusion criteria in the DR-specific conditions, this study excluded individuals with active macular neovascularization or associated macular neovascularization and those who had previously undergone vitrectomy or pan-retinal photocoagulation. Medical history and baseline ocular examinations, including visual acuity, intraocular pressure, ocular structures, fundus imaging, and optical coherence tomography angiography were evaluated in all groups. Additionally, optical coherence tomography-angiography or fluorescein angiography and hematologic blood tests (HbA1c) were performed before injection, according to medical records and National Health Institution requirements. This study complied with the Declaration of Helsinki and was approved by the Institutional Review Board of NTUH (202111013RINB) and the samples were obtained through the informed consent of the participants.

### Tear Fluid Collection and Storage

Tear fluid was collected from both eyes, with only the affected eye used for analysis. Samples were collected with eyes open, under natural conditions, without topical anesthesia, for 5 min or earlier, if dampened.^20^ Tear fluids were sampled around noon to early afternoon using plain Schirmer strips (Haag-Streit, UK). The strips are let dry in the storage Eppendorf tubes and stored at −80 °C until further experiments. Figure 1 shows the experimental flowchart.

**Figure 1:**
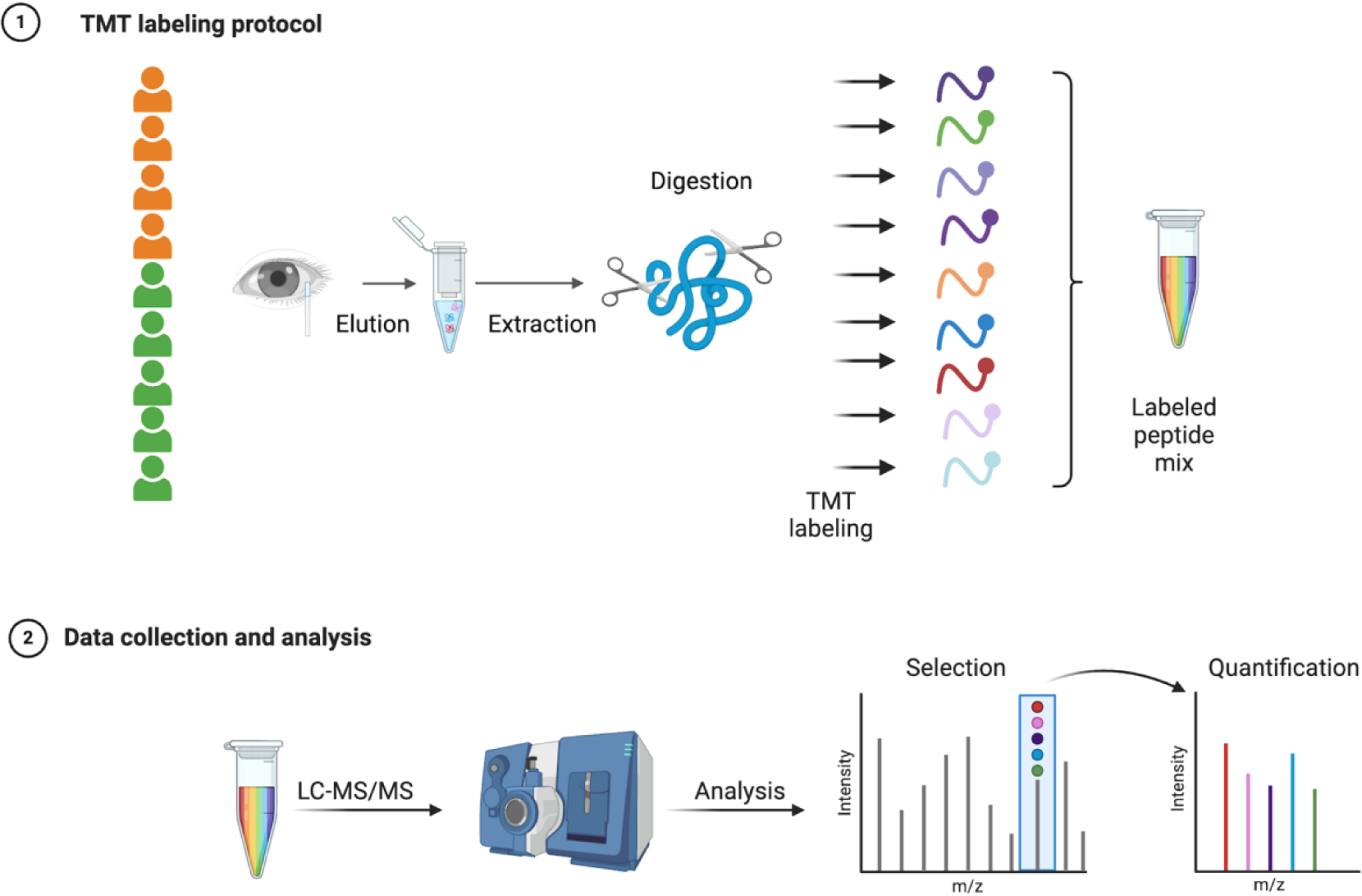
Pipeline for tear fluid proteome study. Tear fluids were extracted with Schirmer’s strip and eluted prior to liquid chromatography–mass spectrometry/mass spectrometry (LC–MS/MS). Each sample was labeled with one specific tandem mass tag (TMT) prior to LC–MS/MS.

### Extraction of the Tear Fluid Proteins

Tear fluid was extracted from strips using 200 μL 100 mM ammonium bicarbonate, and Protease Inhibitor Cocktail (TargetMol, USA) was added to avoid protein degradation. The strips were cut into fragments, thoroughly soaked, and subsequently bathed at 40 °C for 10 min. They were then centrifuged at 12,000 rpm at 4 °C for 10 min. The suspension was then collected for protein analysis.

### Quantitative Tear Fluid Proteomics

Protein samples (20 μg) were prepared for liquid chromatography-tandem mass spectrometry (LC–MS/MS) using tandem mass-tag (TMT) labeling. Following resuspension, the samples underwent in-solution digestion. Proteins were reduced with 5 mM dithiothreitol (DTT), alkylated with 15 mM iodoacetamide (IAA), and digested using Trypsin/Lys-C Mix (Promega, USA). The resulting peptides were desalted using Pierce Peptide Desalting Spin Columns (Thermo Fisher, USA) prior to labeling with TMT reagents (Thermo Fisher, USA). TMT reagents were added to the peptide at a 1 mL:2.5 mg ratio, followed by the addition of 5% hydroxylamine to the solution. Each tear fluid sample was individually labeled with a single tag. The combined precipitate with different tags was reconstituted in a loading buffer (0.1% formic acid) making final protein to 0.3 μg and subjected to LC–MS/MS analysis. Peptide separation was achieved using an Ultimate 3000 nanoLC system (Thermo Fisher Scientific, Bremen, Germany) coupled online to an Orbitrap Fusion Lumos Tribrid quadrupole-ion trap-Orbitrap mass spectrometer (Thermo Fisher Scientific, San Jose, CA). Chromatographic separation was performed on a 75 μm ID, 25 cm length C18 Acclaim PepMap column (Thermo Scientific, San Jose, CA, USA) packed with 2 μm particles and a 100 Å pore size. Mobile phase A consisted of 0.1% formic acid in water, and mobile phase B was 100% acetonitrile with 0.1% formic acid. A segmented gradient was run over 90 min, starting from 2% to 40% solvent B at a constant flow rate of 300 nL/min. The mass spectrometer was operated in data-dependent acquisition mode. Full-MS scans were acquired with a mass accuracy of <5 ppm and a resolution of 120,000 at *m*/*z* = 200, with an automatic gain control (AGC) target of 5e5 and a maximum injection time of 50 ms. The most intense ions were selected for HCD-MS/MS fragmentation for a duration of 3 s. MS/MS spectra were acquired at a resolution of 60,000 within a 1.4 Da isolation window and a normalized collision energy of 36%. An AGC target of 5e4 was set for MS/MS analysis, and previously selected ions were dynamically excluded for 60 s with a maximum injection time of 50 ms.

### Data Processing

Raw MS files were processed using Proteome Discoverer (version 2.4) with Mascot and Sequest HT engines against the SwissProt *Homo sapiens* database (download in 2020.02). The search parameters were as follows: a precursor mass tolerance of 10 ppm and a product ion tolerance of 0.05 Da. The digestion enzyme used was trypsin, allowing up to two missed cleavages. Carbamidomethylation of cysteine (+57.021 Da) was included as a fixed modification, whereas methionine oxidation (+15.995 Da) and acetylation at the protein N-terminus (+42.011 Da) were considered variable modifications. The minimum peptide length was set to seven residues and the false discovery rates (FDRs) of the peptide and protein were both set to 1%. Mass spectrometry proteomic data were deposited in the ProteomeXchange Consortium via the PRIDE partner repository with the data set identifier PXD061821.

### Bioinformatics Analysis

Gene Ontology (GO) term analysis was used to identify enriched biological themes via UniProt.^19^ Gene set enrichment analysis (GSEA; Broad Institute, USA) was conducted to comprehensively analyze the proteins identified by mass spectrometry. When analyzing the proteomics data, we used statistical analysis with the inborn software of previously described toolkits. Statistical significance was set at *p* < 0.05, using permutation-based FDR for compensation. GO,^20,21^ Reactome,^22,23^ and Kyoto Encyclopedia of Genes and Genomes (KEGG) databases were used as reference pathways. Metascape^24^ and WebGestalt (WEB-based Gene SeT AnaLysis Toolkit) (Zhang Lab, Baylor College of Medicine, Houston, Texas, USA) were used for pathway clustering and data presentation, respectively. The figures were refined using R (version 4.3.2).

### Enzyme-Linked Immunosorbent Assay (ELISA)

ELISA was performed to analyze specific proteins in the tear fluids of 21 eyes in 13 patients. Metallopeptidase inhibitor 1 (TIMP-1), matrix metalloproteinase-9 (MMP-9), and insulin-like growth factor-binding protein 3 (IGFBP3) were tested according to the manufacturer’s protocol (R&D Systems, Minneapolis, MN, USA). Equal volume from each tear fluid sample was diluted to 50, 8, and 8 times for TIMP-1, MMP-9, and IGFBP3, respectively. The final volume of the diluent for the assay was 50 μL per well. The absorbance was read at an optical density (OD) of 450 nm using a microplate reader, and 540 nm was used as the background. Concentrations were interpolated using a standard curve.

## Results

### Subject Characteristics

Nine eyes with DME from six patients were included in the tear fluid extraction and proteomic study. Two patients with both eyes enrolled were in the responder group, while one was in the nonresponder group. The status of DR in the enrolled eyes was 5 with PDR and 4 with NPDR. (Supplementary Table S1 shows the demographic details.) We used clinical data to evaluate the treatment response 1 month after anti-VEGF intravitreal injections. Based on the on the reduction in macular thickness after second or third injections, patients were divided into two categories: responders or nonresponders. Nonresponders were defined as those who experienced less than a 20% reduction in macular thickness following the second or third injections, while responders were those who had more than a >20% reduction or complete resolution of fluid. The reductions in central retinal thickness (CRT) were measured as the difference in CRT from baseline and 250 μm.^13,20,21^

According to these criteria, five eyes responded well to anti-VEGF treatment, whereas four were refractory to anti-VEGF injections. Figure 2 illustrates the B-scan OCT images showing responders and nonresponders. In the figure, a subset of DME presenting with diffuse retinal thickness rather than overt intraretinal or subretinal fluid is also shown. This input argues for the diversity of DME and representativeness of the cases.

**Figure 2:**
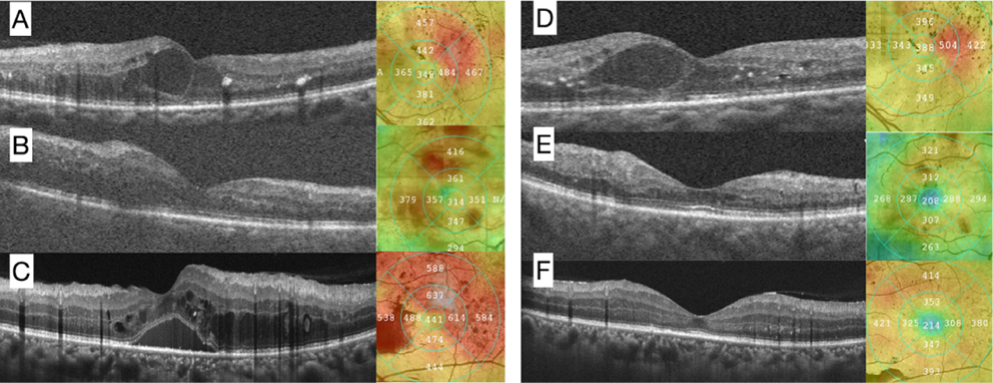
Sample images of optical coherence tomography in diabetic macular edema (DME) subjects. Images A, B, and C are pretreatment scans, while images D, E, F were taken 1 month after the third anti-VEGF injection, corresponding to subjects A, B, and C, respectively. Subject A–D is a typical example of a nonresponder, as indicated by the retention of intraretinal fluid in the images. Additionally, hyperreflective exudates are visible in both A and D. Subject B–E is classified as a responder, exhibiting diffuse edema. Subject C–F is also a responder in DME. In the pretreatment image C, there is a noticeable accumulation of both subretinal and intraretinal fluid, while in F the fluids were not visible anymore.

### Comparative Proteome Analysis on the Tear Fluid of DME

A total of 3121 proteins were quantified in the tear fluid of DME subjects (Supplementary Table S2). The top proteins present in DME tear fluid were involved in gluconeogenesis, maintenance of cellular localization, canonical wingless-related integration site (WNT) pathway, and extracellular matrix organization (Figure 3). This constitution reflects the picture of the targeted disease, and the pathways are in concordance with the pathophysiology of diabetic macular edema. To further differentiate the top proteins between responders and nonresponders, we observed that pathways linked to keratinization and cell membrane and extracellular membrane organization were abundantly expressed in responders, whereas proteins associated with glucose metabolism, response to oxidative stress, and immune response were upregulated in nonresponders.

**Figure 3:**
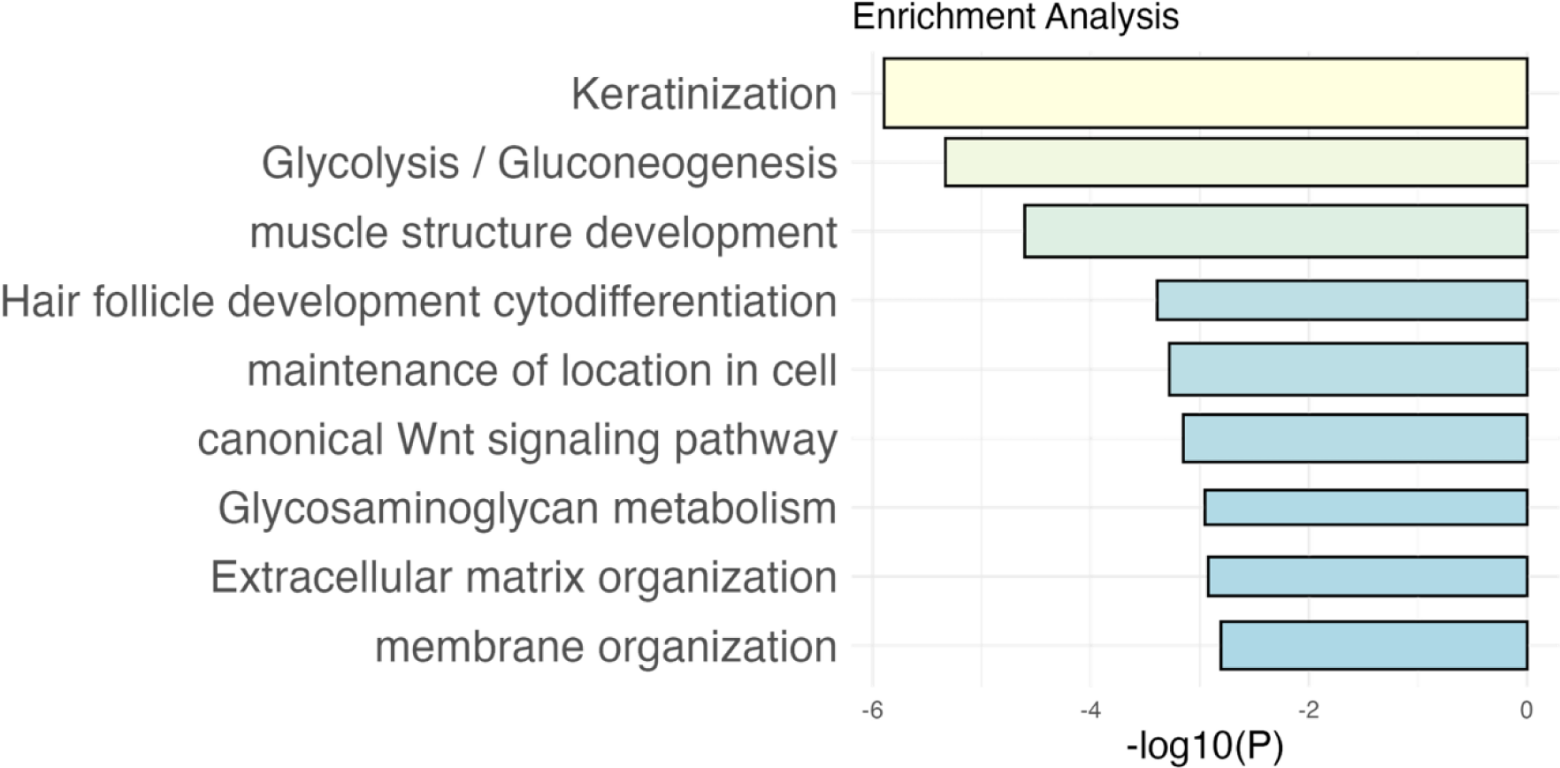
Bar plot of the top enriched protein pathways in diabetic macular edema. Metascape was used as database. The width of the bar associated with the protein numbers involved in the specific pathway; the length of the bar is associated with the significance level (−log *P*).

In particular, MICAL Like 1 (MICALL1) and tyrosine kinase Janus kinase 2 (JAK2) were higher in the tear fluid of nonresponders. MICALL1 is linked to different membrane activities such as protein localization to endosomes and endocytic recycling. Additionally, MICALL1 is linked to age-related macular degeneration during receptor-associated endocytosis.^21^

Additionally, transthyretin (TTR) was upregulated in responders. TTR is a homologous tetramer protein secreted by human RPE cells, choroid tissues, and human retinal microvascular endothelial cells (hRECs) and promotes hREC apoptosis, thereby inhibiting neovascularization.^22^ Subsequent *in vitro* studies showed that TTR counteracted VEGFA, which inhibited hyperglycemia-induced proliferation, migration, and angiogenesis of hRECs, and reversed the DR complication cascade by modulating the VEGFA/PI3K/AKT pathway.^23^

### Differences in Pathway Enrichment in Association with Anti-VEGF Treatment Responsiveness

Proteins included for bioinformatics analysis were required to be present in at least two samples per group. After filtering, 1008 proteins were present (Figure 4A). Additionally, only proteins with a Euclidean distance (calculated from the *p*-value and fold change) >0.6 were considered for hierarchical clustering and principal component analysis (PCA). Following refinement, 156 proteins showed substantial differences in regulation between responders and nonresponders. PCA was used to separate the two groups using filtered proteins. The scattered dots within the shaded ovals indicate some degree of heterogeneity with partial overlap despite the overall distinction between the groups. This phenomenon was expected considering the spectrum of diabetic retinopathy and its coexistence with DME (Figure 4B).

**Figure 4:**
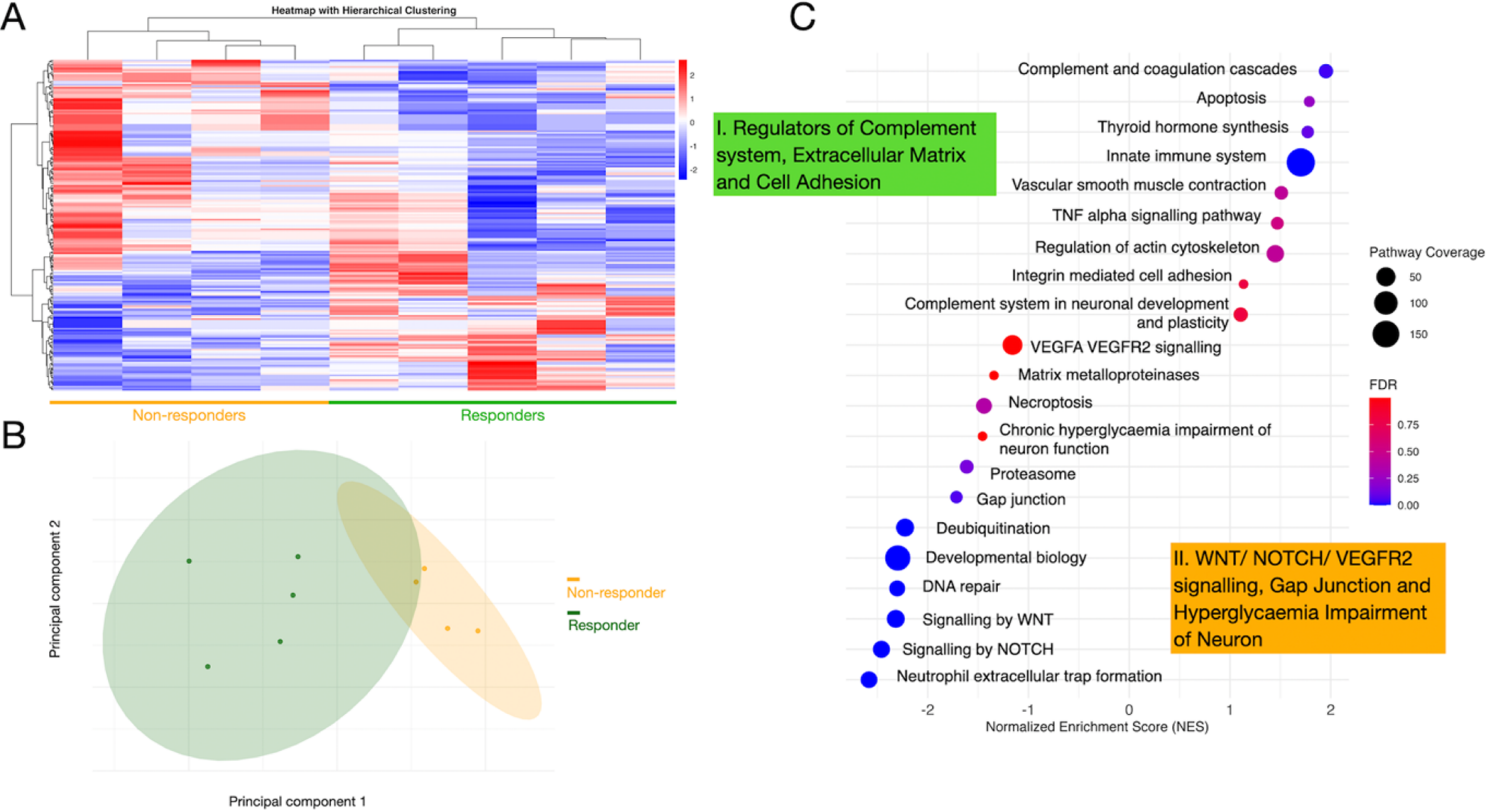
Tear fluid proteome data were extracted for responsiveness analysis. A. The hierarchical heatmap grouping between responders and nonresponders, the color scale reflects the concentration of tear fluid proteins. The proteins used for clustering are those present in at least two samples per group and limited to a Euclidean distance larger than 0.6 (156 proteins in total). B. The visualization of principal component analysis for A protein set (156 proteins in total). C. The plot using gene set enrichment analysis (GSEA) against Reactome, KEGG and Wikipathway database to visualize the enrichment results in distinct responding groups. Proteins included for analysis are those present in at least two samples per group; a total of 1008 proteins were included.

Hierarchical clustering with heatmap visualization effectively distinguished responders from nonresponders (Figure 4A and B). A small subset emerged within the responder group, potentially corresponding to variations in the characteristics of initial edema observed in the OCT images. The middle and bottom images in Figure 2 depict the two edema types included in the study, both of which resolved after anti-VEGF treatment. This subset clustering may provide insight into the influence of initial edema differences on the treatment response. In the current plot, distinct protein clusters were observed, with 85 upregulated and 71 downregulated proteins. Pathway analysis was conducted using GSEA against the Reactome, Wikipathways, and KEGG databases. Pathway analysis was performed without filtering for Euclidean distance, and 1008 proteins were used to avoid overcorrecting the innate weighting system in GSEA. Figure 4C shows the normalized enrichment score and FDR for the characteristic pathways. The complement cascade and extracellular matrix were highly upregulated in responders, along with pathways involved in actin cytoskeleton organization and integrin-mediated cell adhesion. Notably, the role of the complement system in neuronal development and plasticity was enriched within the complement cascade, underscoring the distinctive features of the responders. Conversely, nonresponders showed enrichment in pathways associated with matrix metalloproteases, neutrophil extracellular trap formation in the immune response, and signaling via the VEGFR2, WNT, and NOTCH pathways, all of which are indicative of unresolved edema. Furthermore, chronic hyperglycemia-induced neuronal dysfunction was upregulated in the nonresponders, whereas neuronal plasticity pathways were enriched in the responders.

Differential enrichment of tear fluid proteome pathways aligned with the clinical status of responders and nonresponders. In the responders, most proteins were linked to cellular adhesion, extracellular matrix remodeling, and neuronal plasticity, which is consistent with the pathways supporting resolution and tissue recovery. Contrastingly, nonresponders showed an abundance of proteins associated with destructive processes and active cellular proliferation, reflecting ongoing vascular dysfunction and unresolved edema. These results suggest that the tear fluid proteome profile may serve as a molecular signature for distinguishing treatment responses in DME, with metalloproteinases and WNT and NOTCH signaling playing potential roles in refractory disease.

### Confirmation of Specific Tear Fluid Indicators in Relation to Responsiveness in the Validation Arm

We further evaluated the importance of the pathways mainly affected by responsiveness to anti-VEGF treatment. To validate our findings, we collected tear fluids from an additional 12 eyes belonging to 7 patients. In total, the samples consisted of 10 eyes from nonresponders and 11 eyes from responders, all of which were utilized for the ELISA analysis. Clinical characteristics as Supplementary Table S3.

Matrix metalloproteinases (MMPs) have been linked to DR pathophysiology. They are associated with inflammation and destruction of extracellular matrix (ECM). Among these, MMP-9 is largely produced when NOTCH signaling is activated and is also a well-known inflammatory and destructive ECM target. In responders, the mean value of MMP9 was significantly lower than that in nonresponders, which could imply improve control of the inflammatory status in responders than in nonresponders (Figure 5A, *p* < 0.05).

**Figure 5:**
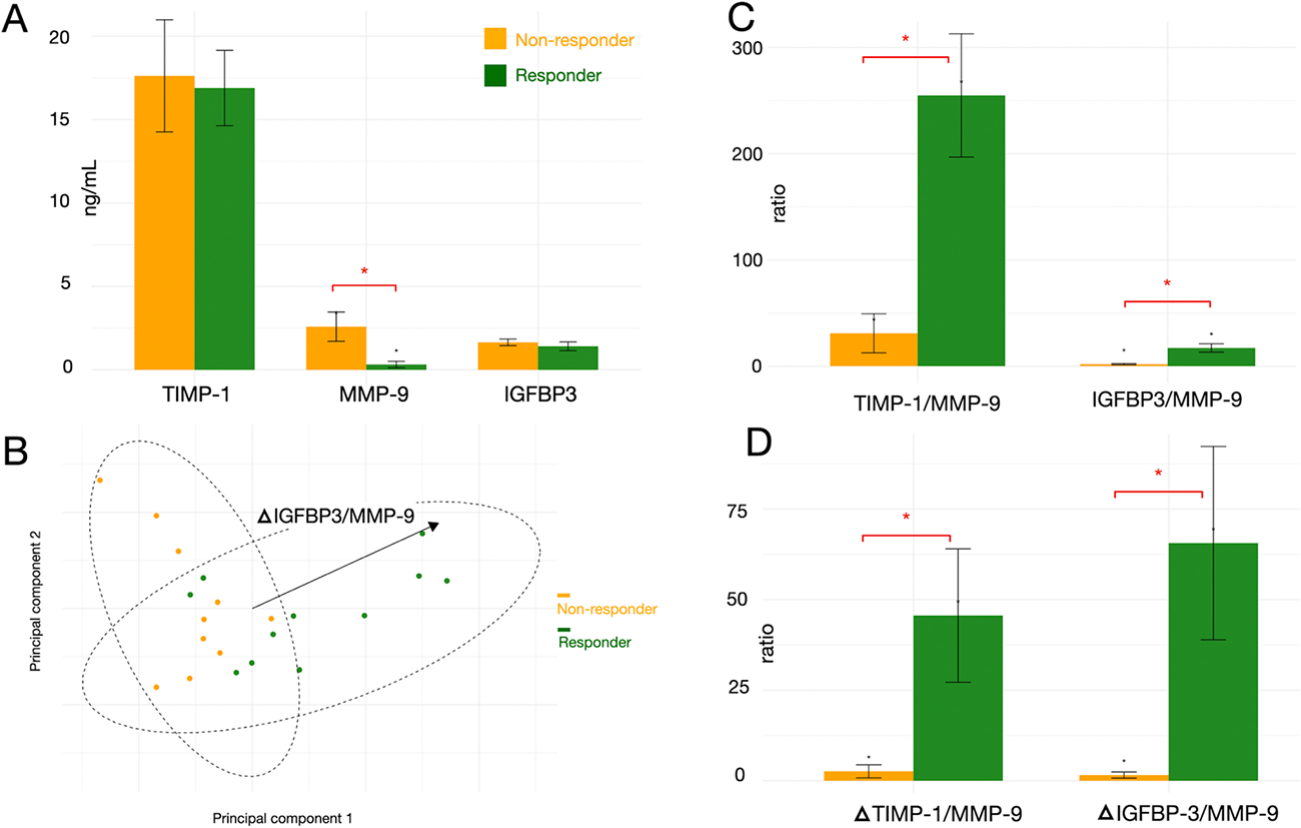
Enzyme-linked immunosorbent assay (ELISA) data of the targeted tear fluid protein samples. A. TIMP-1, MMP-9, and IGFBP3 levels (ng/mL) in different groups. B. The principal component analysis (PCA) plot using bar plot data. The arrow indicates the change of IGFBP3 to MMP-9 ratio was most associated with group difference (treatment responsiveness in this study). C. The ratios of TIMP-1/MMP-9 and IGFBP3/MMP-9 following treatment across various groups. D. Changes in the above ratios, calculated as the difference between post-treatment and pretreatment values, across the same groups. Asterisk (*) denotes statistical significant difference between groups (*p* < 0.05).

MMP activity is tightly regulated by endogenous and TIMP. TIMP-1 is a potent inhibitor of most MMPs, including MMP-1, MMP-2, MMP-3, and MMP-9. TIMP-1 can also bind to pro-MMP-9, blocking enzyme activation of the enzyme.^24^ In various pathological conditions, the balance between TIMP and MMP has been shown to offer more physiologically meaningful insight than isolated measurements. Thus, TIMP-1 was selected for validation. While the mean level of TIMP-1 was not substantially different between groups, the TIMP-1/MMP-9 ratio was significantly higher in responders, suggesting a greater buffering capacity against MMP-mediated inflammation (Figure 5C, *p* < 0.05).

In addition to ECM degradation, pericyte and neuronal health is essential in retinal recovery. IGFBP3, a protective modulator in the retinal microenvironment, was included for validation. Although its absolute level did not differ significantly between groups, the IGFBP3/MMP-9 ratio was significantly elevated in responders (Figure 5C, *p* < 0.05). While IGFBP3 and MMP-9 are not direct biochemical antagonists, their opposing roles in vascular permeability and neurovascular unit stability provide a rationale for this comparison.^25–27^ IGFBP3 has been shown to preserve blood-retinal barrier (BRB) integrity and promote pericyte survival, while MMP-9 disrupts tight junctions and contributes to vascular leakage and neuronal damage. Therefore, this ratio may reflect the relative dominance of neuroprotective versus matrix-degrading processes and serve as a surrogate marker of retinal resilience. Additionally, the change in the ratio of TIMP-1 to MMP9 and insulin-like growth factor binding protein-3 (IGFBP-3) to MMP-9 before and after anti-VEGF treatment was markedly different between responders and nonresponders (Figure 5D). The ratios were higher following treatment in the responders, implying improved retinal resilience and fewer inflammatory and destructive processes.

These three targets, TIMP1, MMP9, and IGFBP3, are also proteins involved in the Reactome pathway, regulating insulin-like growth factor transport and uptake by IGFBP, which were enriched in the GSEA of responders. To evaluate the power of the few targets we tested for, we visualized the results using PCA for tear fluid classification, which corresponds to treatment responsiveness to anti-VEGF, with PC1 achieving statistical significance (*p* = 0.0014) and the axis for the difference between groups coinciding best with the change in IGFBP3 to MMP9 ratio (Figure 5B). In summary, the ELISA targets corresponded to previous pathway analyses and revealed a strong correlation with clinical observations.

## Discussion

This study focused on the tear fluid proteome in DME and identified distinct differences between treatment responders and nonresponders. We observed modulation of the complement cascade and immune response-related pathways as well as cellular adhesion regulation in responders. In nonresponders, tear fluid proteomic pathways were enriched in NOTCH signaling, WNT signaling, and immune responses, such as the formation of neutrophil extracellular traps (Figure 6A and B).

**Figure 6:**
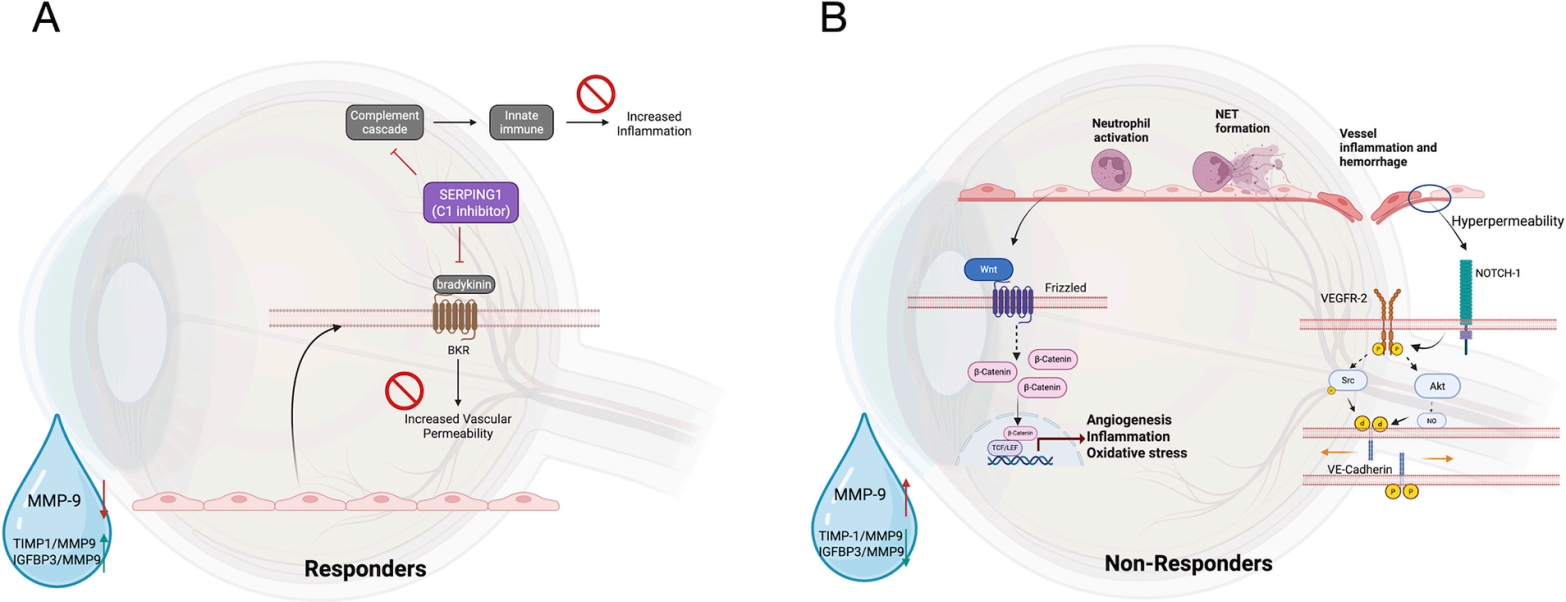
Molecular pathways in tear fluid. A. Pathways enriched in tear fluid proteome of treatment responders and corresponding pathophysiology in illustration. Complement and innate immunity was regulated majorly by SERPING1, a complement 1 (C1) inhibitor. This inhibitor also modulates bradykinin, a potent vasodilator. Proteins in tear fluid depicted the ELISA result of the responders. B. Pathways enriched in the tear fluid proteome of treatment nonresponders and corresponding pathophysiology. The plots illustrate key signaling pathways, including NOTCH, WNT, and subsequent immune and inflammatory response pathways (NETosis in specific) in nonresponders. Proteins in tear fluid depicted the ELISA result of the nonresponders.

In traditional neovascularization, VEGF is essential.^29^ With the introduction of anti-VEGF therapies, the treatment and management of various retinal neovascular diseases have significantly improved.^2,30^ However, in DME, neovascularization is not the sole factor involved; inflammation also plays a crucial role in the disease’s pathophysiology. To further assess disease mechanisms, it is important to know that in DME, neovascularization is not the sole factor involved; inflammation also plays a critical role in the disease pathophysiology.^28–30^

The tyrosine kinase JAK2 regulates several crucial cellular signal transduction pathways. Under hyperglycemic conditions, Ang II mediates the activation of JAK-2 and the downstream pathway.^31^ The resultant effect on the vasculature is chronic inflammation, noticeable in the vascular endothelium.^32^ Adhesive molecule activation that attracts inflammatory cells results in well-known DM-related vasculopathies, along with the blood–retina barrier at the back of the eye.

The NOTCH and WNT pathways contribute to vascular permeability, retinal pigment epithelial cell migration and transformation, and the recruitment of inflammatory cells, all of which exacerbate retinal damage and result in more severe retinopathy.^33,34^ Notably, NOTCH signaling also interacts with MMP-9 accumulation in adjacent tissues, potentially reflecting the disrupted blood–retinal barrier linked to DME (Figure 6B).

In the tear fluid of DME nonresponsive to VEGF, WNT and NOTCH signaling could contribute to refractory disease. WNT signaling is a complex regulatory system that influences cell polarity, proliferation, differentiation, survival, and self-renewal via diverse ligand–receptor interactions.^35^ This pathway plays a crucial role in retinal vascular homeostasis, and its dysregulation contributes to retinal neovascularization.^36^ Notably, the WNT pathway has been implicated in diseased angiogenesis, as patients with DR show decreased DKK1 levels, a natural WNT inhibitor, in plasma and vitreous fluid. This suggests that excessive WNT activity drives uncontrolled endothelial cell proliferation and neovascularization.^37,38^ Additionally, the loss of Fzd7 (a key WNT receptor) inhibits ischemia-induced neovascularization, underscoring its role in endothelial cell pathophysiology.^33^

Similarly, NOTCH signaling is a critical vascular development and angiogenesis modulator, especially in the regulation of endothelial sprouting and tip-stalk cell selection.^39^ Under diabetic conditions, hyperglycemia induces Jagged1 (JAG1) and Delta-like 4 (DLL4) upregulation that activate NOTCH1 and compromise endothelial junctional integrity. While JAG1 and DLL4 do not exert the same potent effects of vascular permeability as VEGFA, their sustained activation in a chronic disease state such as DME may exacerbate vascular dysfunction over time, resulting in persistent leakage and resistance to anti-VEGF treatment.^40^ Furthermore, activation of the NOTCH pathway can stimulate VEGFR2 independently of VEGFA, potentially explaining VEGF nonresponsiveness.

A study on the tear fluid proteome in DR severity conducted in 2022 showed 42 differentially expressed proteins in nonproliferative diabetic retinopathy (NPDR) and PDR compared to normal controls and 26 differentially expressed proteins in PDR compared to normal controls. Substantial alterations were noted in 32 proteins associated with oxidative stress and small extracellular vesicles. In their validation study, NPDR revealed higher concentrations of IL-2, IL-5, IL-18, TNF-, and MMP-2, -3, and -9 than did the controls. In the PDR group, IL-5, IL-18, and MMP-3, -9 concentrations were markedly higher, whereas IL-13 levels were lower compared to that in the controls.^41^ These findings align with those of the current study. However, we further investigated the DME aspect, which was not addressed in their research. As demonstrated in our previous study,^42^ tear fluid proteomic profiles differ significantly between DME patients and nondiabetic controls.^19^ The retina’s disturbed nature is central to DME. In our study, MMP-9 levels were significantly higher in the nonresponder group, as shown in Figure 5A. This suggests that MMP-9 could be a potential biomarker for treatment response in DME.

While only MMP-9 levels in the tear fluid revealed considerable differences in individual protein expression associated with treatment response, the ratio between ECM components and inflammation modulation also served as a potent indicator of disease prognosis. These results align with the proteomic pathway analyses, underscoring the key features of nonresponders to anti-VEGF treatments and guiding future investigations. ELISA further suggested that TIMP-1 and IGFBP-3 could serve as protective factors against retinal damage, warranting future evaluations of retinal resilience (Figure 5D).

Another relevant study for comparison is that of Campochiaro et al., who assessed pro-permeability factors in steroid implant-treated patients, serving as a counterpart to the current study. Although responsiveness to treatment was not directly addressed, their findings indicated that several aqueous proteins such as prolactin, IGFBP-3, and MMP-9 were correlated with edema reduction following treatment.^43^ These targets coincided with our identified tear fluid proteins, and the high agreement level between studies supports our hypothesis that tear fluid proteins can be used to identify treatment responsiveness. The trends noted in pro-permeability factors and prognosis suggest that persistent inflammation and extracellular matrix degradation play crucial roles in treatment nonresponsiveness and that some of the inflammatory and extracellular matrix cascades are not adequately treated, even with steroids.

Tear fluid is the least invasive and the most readily accessible type of ocular fluid. It comprises secretions from surface cells and contributions from the abundant blood vessels present on the ocular surface. These blood vessels carry rich systemic information that reflects local ocular conditions. Tear fluid has been used to study different ocular diseases.^17,44^ In our study, we took this step further using tear fluid as an alternative method for monitoring responsiveness to DME treatment. These findings were promising, as they reflected the pathophysiology of subjects not responding to anti-VEGF treatment, with inflammation and endothelial hyperpermeability conditions surfacing in the proteome composition. In the responder group, extracellular matrix modulation and inflammation regulation were enriched. Although observed in the tear fluid, these alterations could reflect and correlate with retinal conditions, potentially guiding future treatment targets.

This study had several limitations. First, the relatively small cohort size posed a challenge and rendered the pilot study nature of this project. While we aimed to collect tear fluid from subjects with DME, we excluded those who had undergone previous panretinal photocoagulation or vitrectomy because these procedures notably change the local ocular environment beyond diabetes alone. This criterion filtered out several potential subjects. Second, individual variability in tear fluid composition may have introduced a bias. However, this inherent variability reflects real-world conditions and must be acknowledged. Furthermore, the limited tear fluid volume restricted the testing scope, facilitating the validation of only a limited target number. Another limitation was the lack of standard reference values for the tear fluid proteins, as no established normal ranges exist. We mitigated this by standardizing all experimental procedures and analytical methods to minimize bias. Finally, although a fixed volume was employed for protein extraction, variations in total protein content among individuals are likely due to differences in tear production. This variability may influence the concentration values obtained from ELISA, thereby limiting the reliability of direct comparisons. To mitigate this issue, we also reported results using the ratios of selected target proteins, such as TIMP1/MMP9 and IGFBP3/MMP9, which help reduce the impact of fluctuations in total protein yield.

## Conclusion

Tear fluid proteomic analysis appears to be a promising alternative to intraocular sampling for evaluating DME responsiveness, offering insights consistent with aqueous and vitreous proteomes while aligning with clinical prognosis and disease pathophysiology. Our results support further exploration of tear fluid proteins as potential tools for understanding retinal diseases and tailoring therapeutic strategies.

## Supplementary Material



## Notes

Supplementary Table S1: demographic details of subjects enrolled for proteomics research; Supplementary Table S2: list of proteins identified in diabetic macular edema subjects and this list were the basis for hierarchical heatmap, principal component analysis and pathway analysis; Supplementary Table S3: demographic details of subjects enrolled for ELISA research (XLSX)

## Notes

This study was in part presented in ARVO annual meeting (07.May. 2024).

## Abbreviations

GSEA: gene set enrichment analysis
DEPs: differentially expressed proteins
VEGF: vascular endothelial growth factor
DME: diabetic macular edema
